# A clinical next-generation sequencing study on the microbial profiles of asymptomatic apical periodontitis in type 2 diabetic and systemically healthy individuals following adjuvant antimicrobial photodynamic therapy

**DOI:** 10.1007/s00784-026-06763-5

**Published:** 2026-02-09

**Authors:** Zeliha Uğur Aydin, Hulde Korucu, Sevgi Bulak Yeliz, Kadriye Demirkaya, Birsen Can Demirdöğen

**Affiliations:** 1 Department of Endodontics, Henry M. Goldman School of Dental Medicine, Boston University, Boston, MA USA; 2 Department of Endodontics, Gulhane Faculty of Dentistry, University of Health Sciences, Ankara, Turkey; 3 Department of Biomedical Engineering, TOBB University of Economics and Technology, Ankara, Turkey

**Keywords:** Antimicrobial photodynamic therapy, Apical periodontitis, Microbiome, Next generation sequencing, Type 2 diabetes

## Abstract

**Objectives:**

The aim was to evaluate the effect of type 2 diabetes mellitus on the root canal microbiota and its response to antimicrobial photodynamic therapy (aPDT) combined with chemomechanical preparation in asymptomatic apical periodontitis (AP), by next-generation sequencing.

**Materials and methods:**

A total of 22 teeth with a single root and single canal, diagnosed with asymptomatic AP, were included in this study: 11 teeth from patients with T2D (T2D group) and 11 from systemically healthy individuals (Control group). Root canal samples were collected before root canal treatment and after aPDT combined with chemomechanical preparation. Following chemomechanical preparation, canals were incubated with methylene blue for 5 min and photoactivated with a 630 nm LED for 60 s. Root canal samples were collected at two time points—before treatment (Control.Pre, T2D.Pre) and after treatment (Control.aPDT, T2D.aPDT). Following propidium monoazide (PMA) treatment, genomic DNA was isolated using a silica column method and quantified fluorometrically. The V3–V4 regions of the 16S rRNA gene were amplified and sequenced using the Illumina MiSeq platform. Data were processed in QIIME2 with DADA2 for denoising and classified taxonomically using Human Oral Microbiome Database (HOMD). Diversity analyses and statistical evaluations (PERMANOVA and multivariable association analysis using MaAsLin3; FDR-corrected) were performed in R.

**Results:**

A total of 44 root canal samples (Control.Pre, Control.aPDT, T2D.Pre, T2D.aPDT; *n* = 11) were analyzed via 16S rRNA V3–V4 sequencing, yielding 4.6 million high-quality reads and 2,745 Amplicon Sequence Variants (ASVs). Alpha diversity did not differ between pre and post disinfection procedure samples in healthy individuals (*p* > 0.05), whereas a significant reduction in observed ASV richness was detected in the T2D group after the disinfection procedures (*p* < 0.05). The number of ASVs shared between pre and post disinfection procedure samples was lower in the T2D group than in controls. Beta diversity based on unweighted UniFrac distances showed significant shifts after the disinfection procedures in both groups (*p* < 0.05), while weighted UniFrac analyses showed no significant differences (*p* > 0.05). Taxonomic analysis revealed a post disinfection increase in oxygen tolerant taxa and a reduction in obligate anaerobes in both groups, with more pronounced changes in the T2D group.

**Conclusions:**

Following chemomechanical preparation and aPDT, a pronounced restructuring of the root canal microbiota was observed in both healthy individuals and individuals with T2D. In individuals with T2D, the post-disinfection period was characterized by reduced alpha diversity and a lower number of shared ASVs, indicating more limited microbial continuity.

**Clinical relevance:**

The reduced microbial stability observed in individuals with diabetes suggests that systemic metabolic status may affect microbial responses to chemomechanical disinfection combined with aPDT.

## Introduction

Type 2 Diabetes Mellitus (T2D) leads to profound impairments in immune response due to persistent hyperglycemia, thereby increasing susceptibility to infections. In T2D, reduced neutrophil chemotaxis and phagocytic capacity, elevated release of proinflammatory cytokines from macrophages, and delayed tissue regeneration contribute to more severe progression of infections in periapical tissues [1, 2]. In addition to this immunological dysfunction, diabetes has also been reported to induce significant alterations in the oral microbiota, which may exacerbate both the prevalence and severity of apical periodontitis (AP) [3, 4].

In T2D patients, certain endodontic pathogenic bacteria have been reported to be more abundant than in healthy individuals, which may delay the resolution of apical inflammation and adversely affect root canal treatment outcomes [5, 6]. Hyperglycemia has also been reported to promote biofilm formation and resistance mechanisms in these microorganisms [4]. In addition, it has been suggested that chronic apical infections may impair the metabolic control of diabetes and exacerbate insulin resistance by increasing systemic inflammatory markers [7]. These findings suggest that diabetes influences both the development and progression of AP, and conversely, that AP may negatively impact the systemic control of diabetes. Therefore, in diabetic individuals, endodontic disinfection protocols should be approached in a manner that ensures more precise management of microbial diversity and pathogenic load [1, 8].

In this context, antimicrobial photodynamic therapy (aPDT) emerges as a promising adjuvant approach for controlling endodontic infections and accelerating the regeneration process of periapical tissues [9]. aPDT exerts a direct bactericidal effect by targeting bacterial membranes and disrupting biofilm structure through the generation of reactive oxygen species (ROS), which are produced via the interaction of light-activated photosensitizer agents with molecular oxygen [9]. aPDT has been reported to effectively eliminate various microorganisms that are more prevalent in diabetic individuals and exhibit resistance to conventional treatment protocols [10, 11]. In addition, the low intrinsic cytotoxicity of aPDT, unlike conventional irrigants, may promote the healing of periapical tissues without contributing to the cytotoxic burden. This minimal toxicity profile could play a compensatory role in enhancing tissue regeneration, particularly in systemic conditions such as diabetes, where delayed healing is frequently observed [12]. In this context, the integration of aPDT into endodontic disinfection protocols for diabetic patients has the potential to reduce microbial load and accelerate periapical tissue healing [13].

Our literature review revealed that no next-generation sequencing (NGS)-based analysis has been reported regarding the root canal microbial profile of apical periodontitis following aPDT in either healthy individuals or patients with type T2D. In this study, changes in the microbial composition of root canal samples obtained from T2D and healthy individuals were evaluated using NGS.The null hypothesis (H₀) of the study posits that aPDT does not significantly alter the root canal microbial composition, as assessed by NGS, in either T2D patients or healthy controls.

## Materials and methods

### Subjects

This cross-sectional interventional study protocol was approved by the Scientific Research Ethics Committee of University of Health Sciences, in compliance with the Declaration of Helsinki (Ethics No: 2023–07), and was registered in the Clinical Trials Registry (ID: NCT06931678). Participants were recruited from patients referred to the Department of Endodontics at the Faculty of Dentistry, University of Health Sciences, and were diagnosed with asymptomatic apical periodontitis requiring root canal treatment.

Participants included in the study were categorized into two groups based on their systemic health status: the T2D group consisted of individuals diagnosed with T2D, while the control group included individuals without any systemic disease. Both groups received aPDT as part of the endodontic treatment protocol.

#### T2D group

This group included 11 teeth with AP from 11 individuals (Mean age: 54.0 ± 4.6 years) diagnosed with T2D who had no other systemic conditions. Patients in the T2D group had been previously diagnosed in accordance with the diagnostic criteria of the American Diabetes Association [14] and were selected based on meeting specific metabolic parameters. Inclusion required either fulfillment of at least two diagnostic criteria or the repetition of one criterion on different days. All T2D participants were instructed to continue their current diabetes management regimens (including oral antidiabetic medications), diets, and lifestyles without any modifications throughout the study period.

#### Control group

This group included 11 teeth with AP from 11 individuals (Mean age: 48.2 ± 3.9 years) without any systemic diseases.

Sample size was determined a priori using G*Power software (version 3.1.9.7, Universität Düsseldorf, Germany). Based on effect size estimates derived from previously published clinical studies evaluating changes in Shannon diversity following root canal disinfection (dz = 1.12), a paired-samples t-test model was applied for within-group pre–post comparisons. Assuming a one-tailed significance level of α = 0.05 and a target statistical power of 0.95, the minimum required sample size was calculated as 11 subjects per group. With this sample size, the achieved statistical power was 0.96 [15].

All clinical assessments and endodontic treatment procedures were performed by a single clinician (XX) with three years of experience in a postgraduate endodontic residency program.

### Clinical criteria

Teeth were included if they exhibited pulp necrosis and asymptomatic apical periodontitis (AP), had adequate coronal structure for definitive restoration, and were confirmed radiographically to be single-root and single-canal. Clinical assessment involved vertical and horizontal percussion of the affected and adjacent teeth, palpation of surrounding tissues, and evaluation for swelling or abnormal mobility. Pulpal status was determined using thermal testing (Endo-Ice, Hygenic; Coltene, Switzerland) and electric pulp testing (EPT), and teeth that showed no response to both tests were confirmed as nonvital. Periapical status was assessed radiographically, and only single-root, single-canal teeth with mature apices and a periapical index (PAI) score of 3, 4, or 5 were included [16]. Teeth were excluded if they exhibited marginal periodontitis (probing depths > 3 mm with ≥ 2 mm attachment loss in more than 30% of sites), detectable sinus tracts, direct communication between the pulp chamber and the oral cavity, or radiographic evidence of open apices, canal calcifications, vertical root fractures, root perforations, or internal/external resorption. Teeth for which adequate rubber dam isolation could not be achieved were also excluded.

### General health criteria

Participants aged 30–65 years with a confirmed diagnosis of Type 2 Diabetes Mellitus (T2D) and an HbA1c ≥ 6.5% were assigned to the T2D group, whereas systemically healthy individuals with HbA1c < 5.7% and fasting blood glucose (FBG) < 100 mg/dL after ≥ 8 h of fasting were assigned to the control group. On the day of treatment, a capillary random blood glucose measurement was obtained to ensure procedural safety; T2D participants proceeded if the value was ≤ 300 mg/dL, while control participants with glucose levels > 126 mg/dL were excluded to avoid possible undiagnosed diabetes. Baseline glycemic indices were as follows: in the T2D group, HbA1c = 7.8 ± 1.1% (range 6.5–10.2) and FBG = 154 ± 28 mg/dL (range 108–212); in controls, HbA1c = 5.2 ± 0.3% (range 4.6–5.6) and FBG = 88 ± 7 mg/dL (range 72–98). Individuals with systemic diseases other than T2D, pregnant women, those with a history of head or neck radiotherapy, individuals who had received antibiotics or immunosuppressive therapy within the previous three months, and smokers were excluded to limit systemic confounding.

### Treatment protocol and sampling procedure

Clinical procedures were standardized for both groups, and to eliminate inter-operator variability, all treatments were performed by a single clinician. In each patient, the target tooth was anesthetized and isolated with a rubber dam. For local anesthesia, 2% lidocaine hydrochloride (HCl) with 1:100,000 epinephrine was administered via infiltration. Caries-affected dentin was removed using a sterile round bur (H1 Round Tungsten Carbide Operative Bur; Komet Brasseler, Lemgo, Germany) mounted on a high-speed rotary handpiece, and a conventional access cavity was prepared. In the present study, a multistep coronal disinfection protocol comparable to those previously reported in molecular endodontic studies was applied. Briefly, the operative field was disinfected using cotton pellets moistened with 3% hydrogen peroxide, followed by 2.5% sodium hypochlorite (NaOCl), and subsequently neutralized with 10% sodium thiosulfate and rinsed with sterile saline. This disinfection sequence was performed both before pulp chamber access and after preparation of the access cavity to minimize the risk of coronal contamination prior to microbiological sampling [17, 18]. The root canal was then filled with sterile saline, and the working lengths were determined using #15 sterile hand files (Dentsply Maillefer, Tulsa, OK, USA) and an electronic apex locator (Propex Pixi; Dentsply Maillefer, Ballaigues, Switzerland).

The root canals were irrigated thoroughly with sterile saline, and prior to sampling, they were enlarged to the appropriate working length using a #25 K-type file. Subsequently, 3 to 5 sterile #25 paper points were placed into each root canal and left in place for 1 min to collect baseline microbial samples (Control.Pre: samples collected before disinfection and aPDT in the control group; T2D.Pre: samples collected before disinfection and aPDT in the T2D group). Root canals were shaped using Reciproc Blue systems (#R40 or #R50; VDW, Munich, Germany) depending on individual case anatomy. When a #20 K-file (VDW) could reach working length, the canal was considered of medium size and enlarged to #R40. If a #30 K-file (VDW) could easily reach the working length, the canal was considered wide and instrumented up to #R50 [19]. All canals were irrigated with a total of 10 mL of 2% NaOCl (Cerkamed), followed by 2.5 mL of EDTA (Cerkamed). After completion of chemomechanical preparation, the canals were dried with sterile paper points.

### aPDT procedure

To minimize the risk of tooth discoloration prior to the aPDT procedure, a bonding agent was applied to the access cavities, covering approximately 1 mm below the enamel–cementum junction [20]. Following isolation, all root canals were completely filled with 0.01% methylene blue (Merck KGaA, Darmstadt, Germany), which served as the photosensitizer. The dye was allowed to remain in the canal for a 5-min pre-irradiation period to ensure adequate penetration into the dentinal tubules. For photoactivation, an Endo tip with a 1 mm^2^ emission surface, attached to a Fotosan LED device (Light PDT 630; Easyinsmile International Corp., New Jersey, USA), was used. The LED lamp emitted light within the 620–640 nm wavelength range (85% output), with a peak wavelength of 630 nm, and an intensity of 2–4 mW/cm^2^ for 60 s. The tip was inserted into the root canal and moved slowly in a helical apical to coronal motion to ensure uniform irradiation of the canal walls. All aPDT procedures were performed in a single session. After photoactivation, the remaining methylene blue was flushed out using 2 mL of sterile saline [21]. Post-aPDT sampling was repeated using the same procedure described previously, this time with sterile #40 or 50 paper points. All samples were placed into Nunc CryoTube cryogenic vials (Merck, Darmstadt, Germany) containing phosphate-buffered saline (PBS) as the transport medium. The samples were then transferred into liquid nitrogen and stored in an upright position at − 80 °C until DNA extraction. Root canals were obturated using a resin-based root canal sealer (Dia Dent, Seoul, Korea) in accordance with the lateral condensation technique. Permanent restorations were completed in the same session using direct composite resin (Tokuyama, Tokyo, Japan).

### Genomic DNA extraction

All specimens were transferred on dry ice for DNA extraction. Each root canal sample was vortexed thoroughly prior to use, and 200 µL of the suspension was transferred into a new microcentrifuge tube. Then, 30 µM of propidium monoazide (PMA) solution was added to each sample and incubated in the dark for 10 min [3, 22]. After incubation, the samples were exposed to a LED light source for 20 min to enable cross-linking with free-floating DNA in the environment. The use of PMA allowed selective suppression of DNA originating from dead cells or free extracellular DNA, thereby ensuring that only DNA from viable microorganisms would be amplified and analyzed. Subsequently, genomic DNA was extracted using the Roche® High Pure PCR Template Preparation Kit (Cat. No: 11 796 828 001, USA). A volume of 200 µL Tissue Lysis Buffer was added to the PMA-treated sample. The mixture was supplemented with 15 mg of glass beads and 10 zirconia beads, followed by mechanical homogenization using a bead beater at 4000 rpm for 2 × 20 s cycles. After homogenization, 40 µL of Proteinase K was added, and the samples were incubated at 56 °C for 60 min. The lysate was then centrifuged at 5000 × g for 5 min, and the supernatant was transferred to a new tube. Subsequently, 200 µL of Binding Buffer was added, and the mixture was incubated at 70 °C for 10 min. After incubation, 200 µL of absolute ethanol was added, and the entire volume was loaded onto the spin column provided with the kit. The column was centrifuged at 8000 × g for 1 min and transferred to a new collection tube. Then, 500 µL of Inhibitor Removal Buffer was added to the column and centrifuged at 8000 × g for 1 min, after which the collection tube was replaced. The column was washed twice using 500 µL of Wash Buffer in each wash step. After the final wash, the column was placed into a clean 1.5 mL microcentrifuge tube. A total of 200 µL of Elution Buffer was added to the column, incubated at room temperature for 1 min, and centrifuged to elute the genomic DNA. The concentration and purity of the extracted genomic DNA were evaluated prior to sequencing using the Quant-iT™ PicoGreen dsDNA Assay Kit (Invitrogen, Cat. No: P7589).

### Amplification and sequencing of the V3–V4 regions of the 16S rRNA gene

To characterize the microbial communities, the hypervariable V3–V4 regions of the 16S ribosomal RNA (rRNA) gene—approximately 467 base pairs [2] in length—were targeted from genomic DNA extracted from root canal samples. Amplification was performed using the 16S Metagenomic Library Preparation Kit (Cat. No: 15044223, Illumina, USA) according to the manufacturer’s protocol.

The primer pair used for PCR amplification consisted of universal sequences that are widely employed in microbiome studies. The primers used were as follows:

- 16S Amplicon PCR Forward Primer (515F): 5′-TCGTCGGCAGCGTCAGATGTGTATAAGAGACAG**CCTACGGGNGGCWGCAG**−3′
- 16S Amplicon PCR Reverse Primer (806R): 5′-GTCTCGTGGGCTCGGAGATGTGTATAAGAGACAG**GACTACHVGGGTATCTAATCC**−3′

The bolded regions in the sequences represent the specific binding regions targeting the hypervariable V3–V4 sites of the 16S rRNA gene; while the remaining sequences correspond to adapter regions compatible with the Illumina sequencing platform. For each sample, a total of 10 ng of genomic DNA was added as the template in the PCR reaction mixture. PCR amplification was performed under the following thermal cycling conditions: initial denaturation at 95 °C for 3 min; 25 cycles of denaturation at 95 °C for 30 s, annealing at 55 °C for 30 s, and extension at 72 °C for 30 s; followed by a final extension at 72 °C for 5 min. Verification of PCR amplification products was carried out using 1.4% agarose gel electrophoresis. Quantification of the purified products was performed using a Qubit® 4.0 Fluorometer (Thermo Fisher Scientific, Carlsbad, CA, USA). Dual indexing was performed using the Nextera XT Index Kit (Illumina), allowing each sample to be uniquely barcoded for multiplexing sequencing. Indexed libraries were normalized to equal concentrations and pooled prior to sequencing. Sequencing was carried out on the Illumina MiSeq platform using the MiSeq Reagent Kit v3 (600-cycle), following a 2 × 250 bp paired-end sequencing protocol. Samples were loaded into designated wells provided within the kit, and sequencing was performed automatically by the instrument. Additionally, an extraction negative control was included in each batch of samples to monitor for potential contamination. During both DNA extraction and amplification steps. These controls were processed in parallel with test samples and underwent both PCR and sequencing.

### Microbial community analysis using QIIME2 and R

Raw sequencing data in FASTQ format, obtained from the Illumina MiSeq platform, were processed using the QIIME2 pipeline (version 2023.2; Quantitative Insights Into Microbial Ecology 2; https://qiime2.org/) for the analysis of microbial diversity. Prior to downstream processing, initial quality assessment was performed based on sequencing depth and Phred quality scores to ensure suitability. Based on this quality assessment, the data underwent quality filtering and trimming. During the quality filtering step, the DADA2 (Divisive Amplicon Denoising Algorithm 2) plugin was employed to remove low-quality reads (Phred Q score < 30), chimeric sequences, and other artifacts [23]. Additionally, potential contaminant sequences were identified and removed during data analysis using the R package decontam, based on the frequency and prevalence of sequence features [24]. As a result of this process, high-resolution and quality-filtered sequences known as Amplicon Sequence Variants (ASVs) were obtained. Based on the DADA2 outputs, phylogenetic trees were constructed within the QIIME2 environment using MAFFT for sequence alignment and FastTree for tree building, which facilitated downstream phylogenetic diversity analyses. For taxonomic classification, the Human Oral Microbiome Database (HOMD, version 16.03; https://www.homd.org/ftp/16S_rRNA_refseq/HOMD_16S_rRNA_RefSeq/V16.03/) was employed. ASVs were classified using a naïve Bayes classifier trained on the HOMD reference sequences corresponding to the V3–V4 region of the 16S rRNA gene.

### Statistical analysis

The analysis files obtained from the QIIME2 environment (table-dada2.qza, rooted-tree.qza, taxonomy.qza, and sample-metadata.tsv) were imported into RStudio (version 4.3.1) for advanced statistical analysis and data visualization. These files were integrated using the phyloseq package (version 1.44.0) to construct a phyloseq object. Alpha diversity analyses were conducted using the microbiome package to assess species richness and evenness within individual samples. Differences in alpha diversity among groups were statistically evaluated using the Kruskal–Wallis rank sum test. For beta diversity analysis, inter-sample microbial community dissimilarities were calculated using Bray–Curtis, Jaccard, Weighted UniFrac, and Unweighted UniFrac distance metrics. The statistical significance of beta diversity differences among groups was assessed using the PERMANOVA (Permutational Multivariate Analysis of Variance) test implemented via the Adonis function in the vegan package. To identify differentially abundant taxa across groups, differential abundance analysis was performed using the DESeq2 package (version 1.48.0). Additionally, to detect biologically meaningful microbial biomarkers distinguishing the study groups, LEfSe (Linear Discriminant Analysis Effect Size) analysis was applied, and statistically significant taxonomic features were identified [25]. Differentially abundant taxa were identified using MaAsLin3 (https://huttenhower.sph.harvard.edu/maaslin/),which applies multivariable linear modeling frameworks with appropriate normalization and covariate adjustment to identify taxa associated with study groups. Features with low prevalence (< 10%) were filtered out prior to model fitting. p-values were adjusted for multiple comparisons using the Benjamini–Hochberg false discovery rate (FDR) correction method.

## Results

### Bacterial diversity and richness

**Fig. 1:**
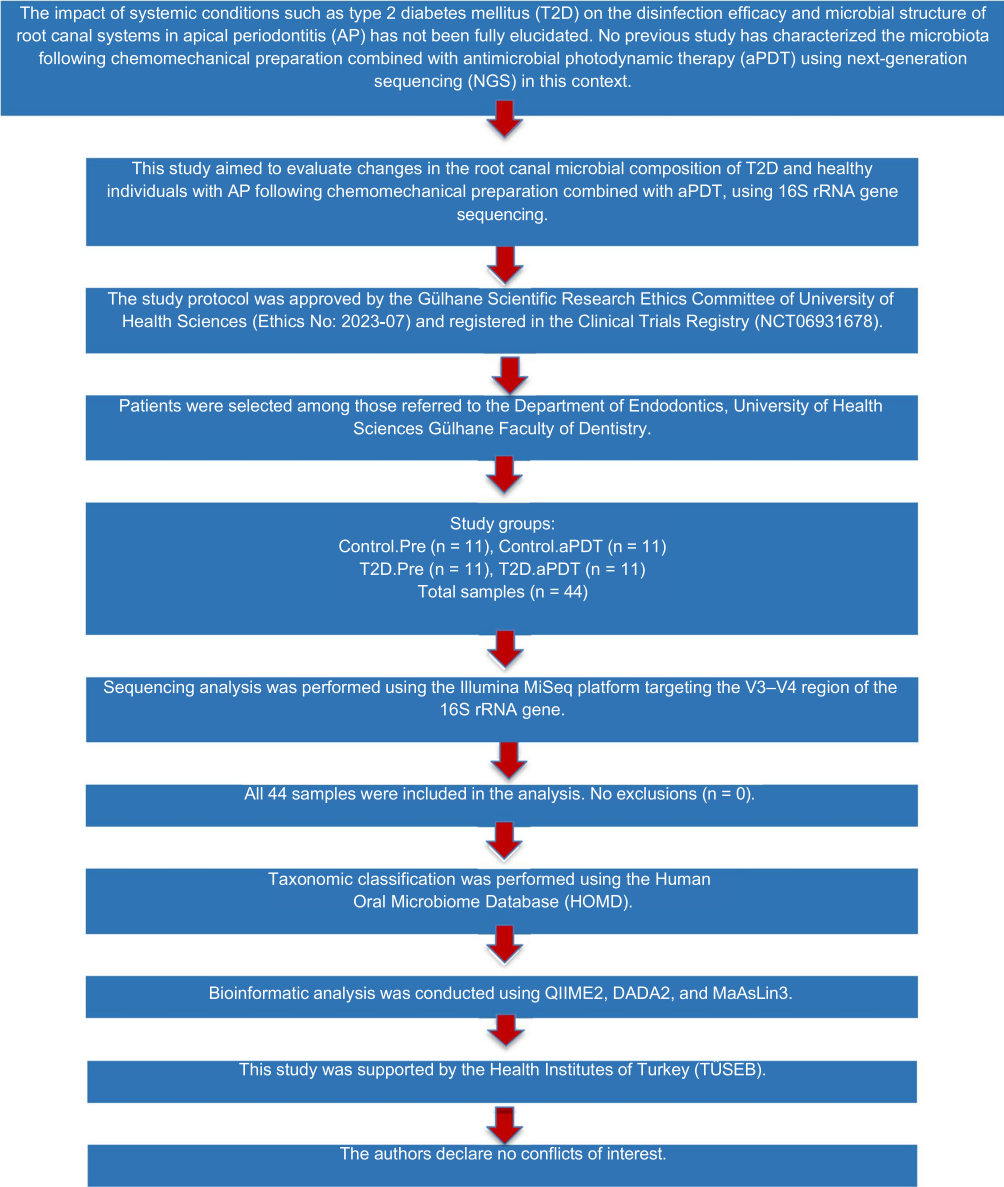
Workflow diagram

Patient selection and experimental procedures are presented in Fig. 1. According to the 16S rRNA gene amplicon sequencing analysis, a total of 44 samples were sequenced from two groups, including pre- and post-disinfection stages with and without aPDT: Control.Pre, Control.aPDT, T2D.Pre, and T2D.aPDT. Each group consisted of 11 samples. Sequencing of the V3–V4 region of the 16S rRNA gene yielded a total of 4,601,414 reads. The average read count per sample was 104,577, with a range between 83,658 and 119,357. The average nucleotide length was 414 base pairs, and the mean GC content was 53.34%. Only reads with a Q30 quality score of 95 percent or higher were included in the analysis.

**Fig. 2:**
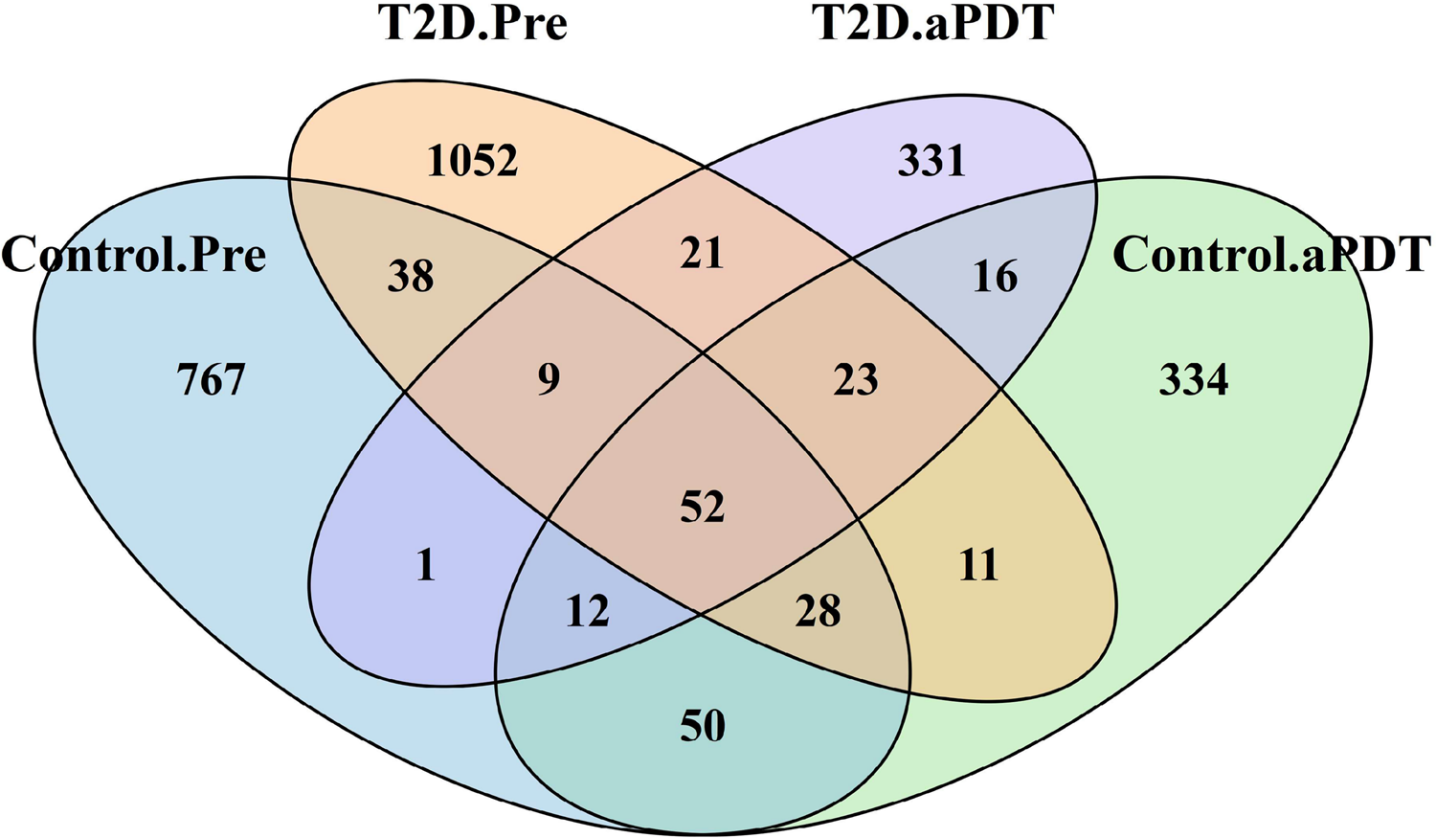
Venn diagram of bacterial ASVs shared among Control.Pre, Control.aPDT, T2D.Pre, and T2D.aPDT groups

**Fig. 3:**
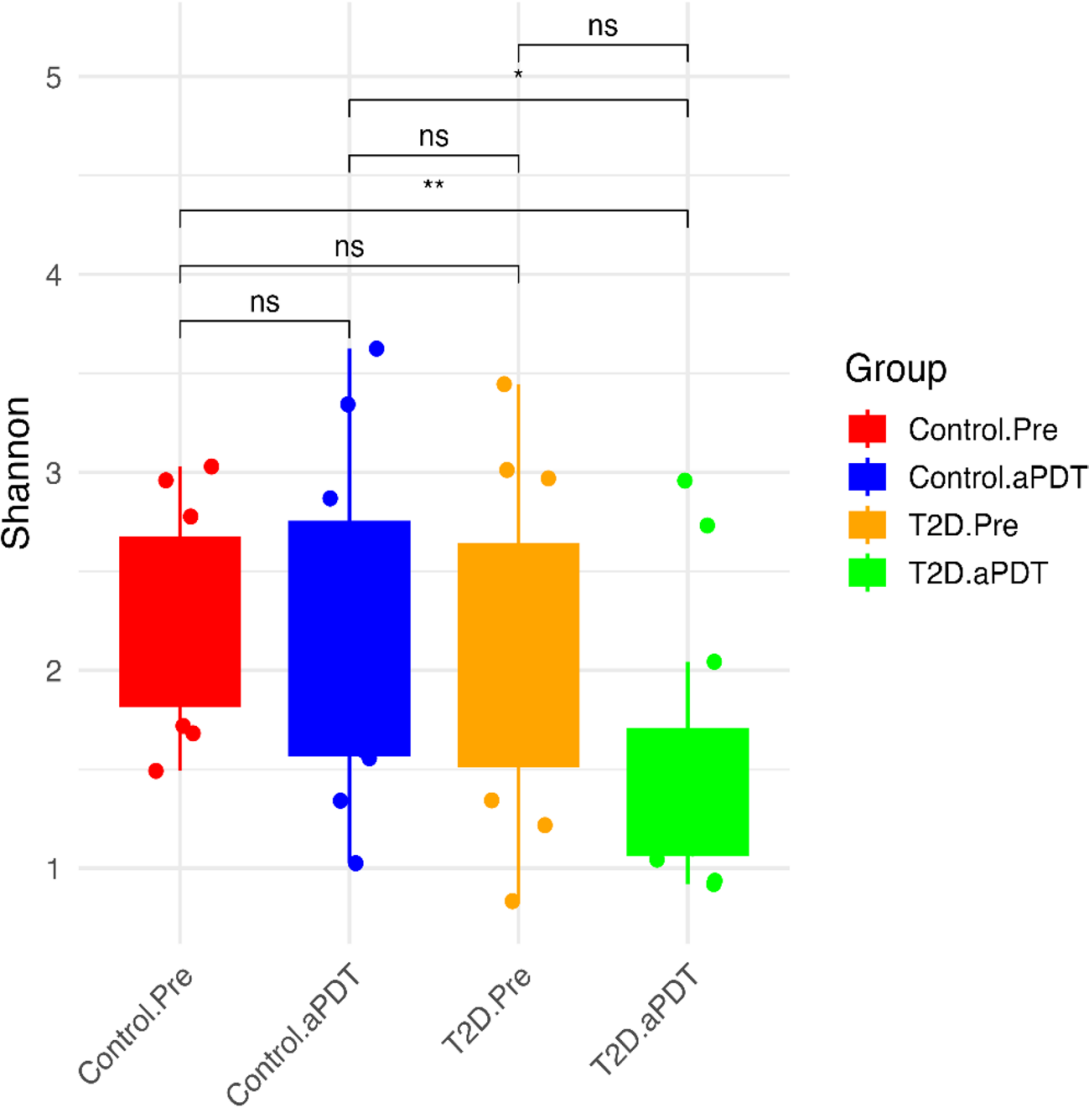
Shannon alpha diversity analysis illustrates the comparison of Shannon diversity indices among root canal samples across the Control.Pre, Control.aPDT, T2D.Pre, and T2D.aPDT groups. Box plots represent the median and interquartile range (IQR), whiskers indicate the minimum–maximum values, and dots denote individual samples. Statistical significance in group comparisons is indicated by ns (not significant), * (*p* < 0.05), and ** (*p* < 0.01)

A total of 2,745 ASVs were identified, and their distribution is illustrated in Fig. 2. In the control group, 1,151 group-specific ASVs were detected; of these, 767 ASVs were present in the Control.Pre samples and 334 ASVs in the Control.aPDT samples, with 50 ASVs shared between both time points. In the T2D group, 1,404 group-specific ASVs were identified, including 1,052 ASVs in the T2D.Pre samples and 331 ASVs in the T2D.aPDT samples, with 21 ASVs shared between both time points (Fig. 2). Alpha diversity was assessed using the observed ASV count and the Shannon diversity index to evaluate microbial community richness and diversity. In the control group, no statistically significant differences were detected between pre-treatment (Control.Pre) and post-treatment (Control.aPDT) samples in either the observed ASV count (*p* = 0.071) or the Shannon index (*p* = 0.797) (Fig. 3A, B). In contrast, a pronounced reduction in alpha diversity was observed in the T2D group following treatment. Specifically, the observed ASV count was significantly lower in the T2D.aPDT group compared with the T2D.Pre group (*p* = 0.00016). Although the Shannon diversity index also showed a decreasing trend after treatment, this difference did not reach statistical significance (*p* = 0.076) (Fig. 3C, D).

### Principal coordinate analysis

**Fig. 4:**
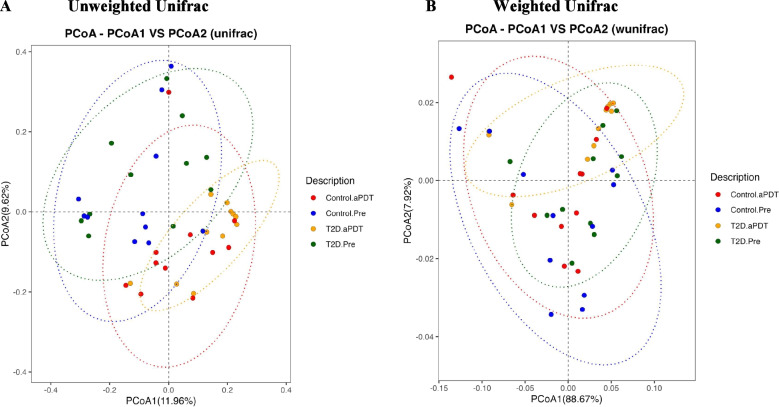
Principal coordinates analysis (PCoA) based on unweighted (**A**) and weighted (**B**) UniFrac distances was used to evaluate beta diversity among study groups. The unweighted UniFrac analysis considers only the presence or absence of taxa, whereas the weighted UniFrac also accounts for relative abundances. Distinct clustering patterns were observed across the four groups: Control.Pre (red), Control.aPDT (blue), T2D.Pre (green), and T2D.aPDT (orange), each represented by different colors and shapes on the PCoA plots

Beta diversity analysis was performed to evaluate overall differences in microbial community structure, and Principal Coordinates Analysis (PCoA) based on unweighted and weighted UniFrac distances was applied for this purpose (Fig. 4A, B). According to the PERMANOVA results, Control.Pre and Control.aPDT groups showed a statistically significant difference in the unweighted UniFrac analysis (R^2^ = 0.07695, *p* = 0.006), whereas no significant difference was detected in the weighted UniFrac analysis (R^2^ = − 0.001805, *p* = 0.976).Within the T2D group, comparison of T2D.Pre and T2D.aPDT samples revealed a statistically significant difference in the unweighted UniFrac analysis (R^2^ = 0.106166, *p* = 0.001), while the weighted UniFrac analysis did not reach statistical significance (R^2^ = 0.064442, *p* = 0.277). For between-group comparisons, no statistically significant difference was observed between Control.Pre and T2D.Pre samples in either the unweighted (R^2^ = 0.040033, *p* = 0.086) or weighted UniFrac analyses (R^2^ = 0.019271, *p* = 0.235). In contrast, comparison of Control.aPDT and T2D.aPDT samples demonstrated a statistically significant difference in the unweighted UniFrac analysis (R^2^ = 0.073784, *p* = 0.028), whereas the weighted UniFrac analysis again showed no significant difference (R^2^ = 0.026946, *p* = 0.183).

### Taxonomic analysis of microbiota

**Fig. 5:**
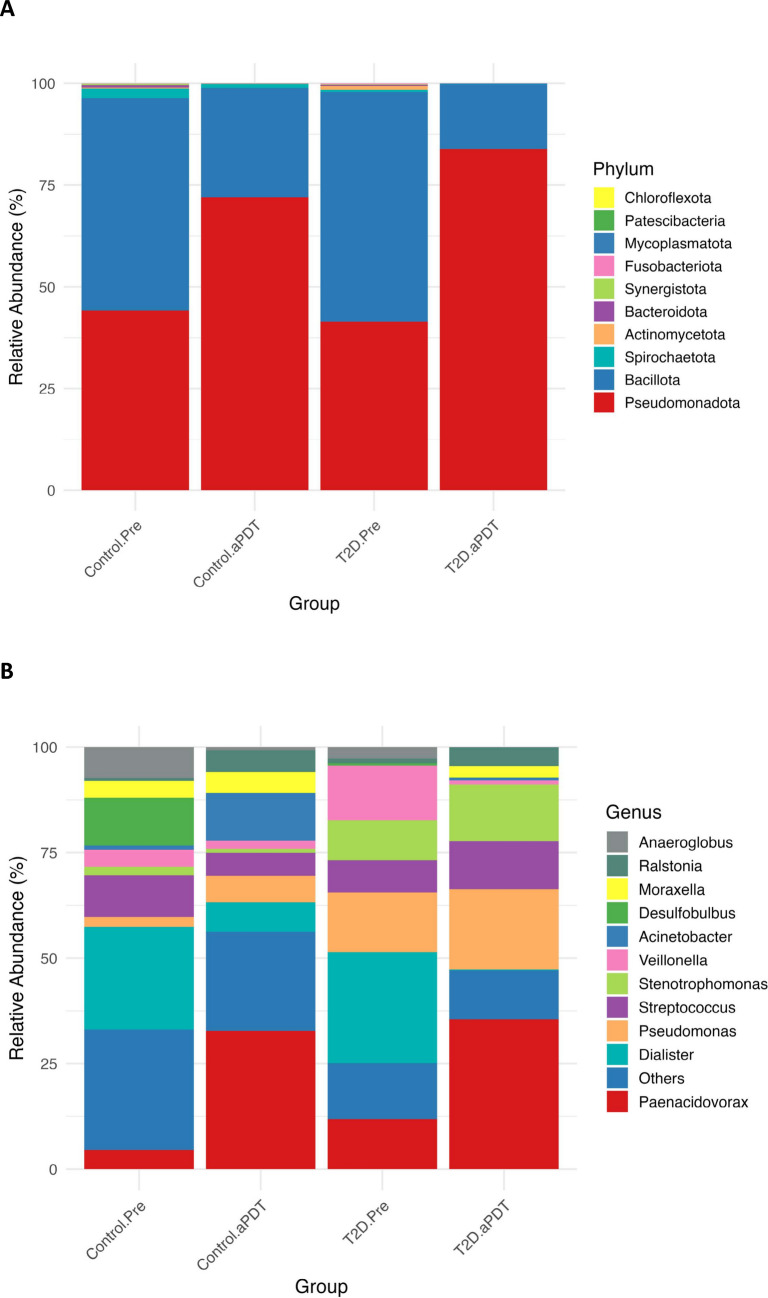

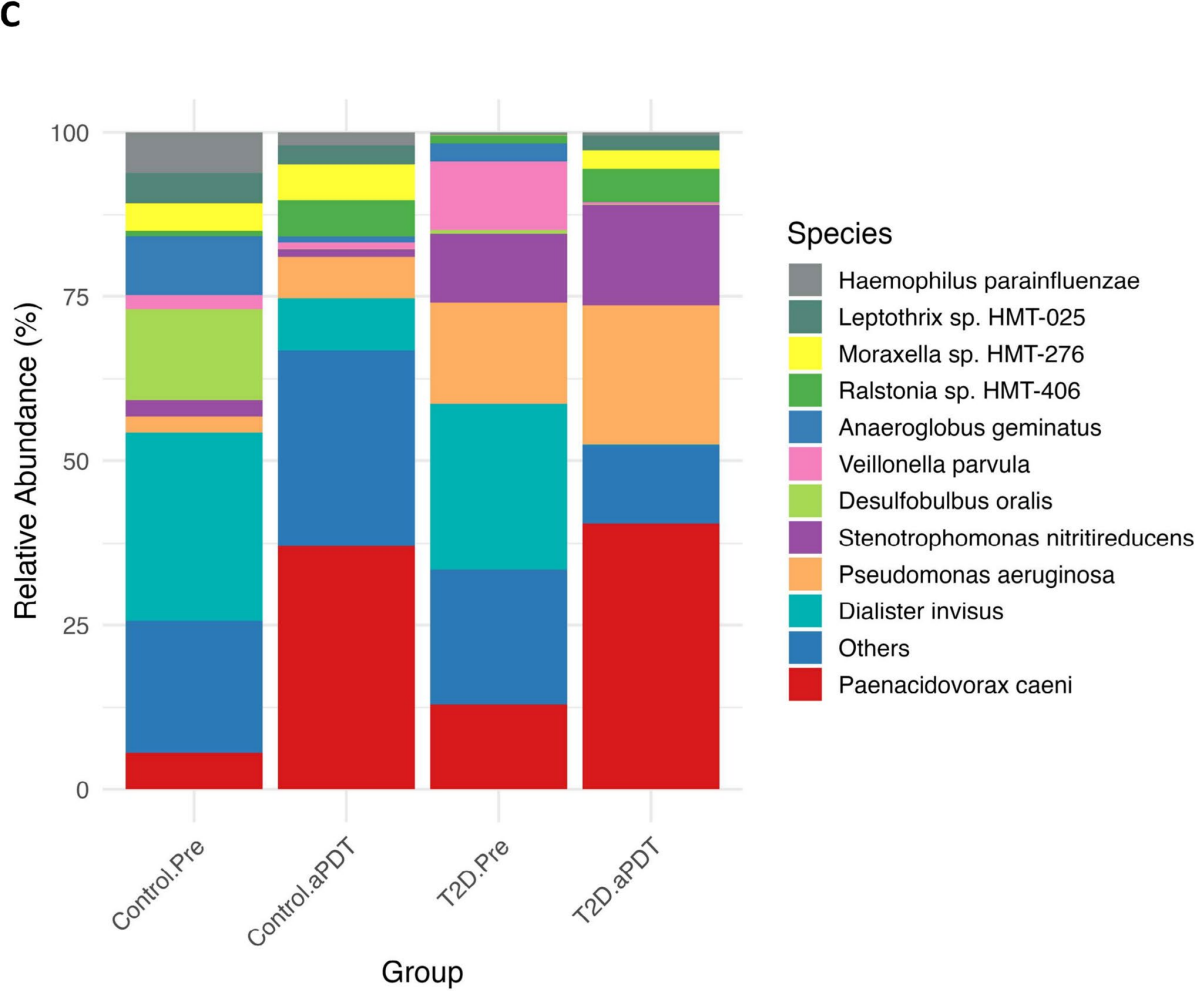
Relative abundances of bacterial communities in the Control.Pre, Control.aPDT, T2D.Pre, and T2D.aPDT are presented. Bacterial taxa are classified at the phylum (**A**), genus (**B**), and species (**C**) levels. Taxa with relative abundances below 1% in each group are collectively categorized as "Others."

Microbial profiles from all samples were taxonomically classified using the Human Oral Microbiome Database (HOMD, version 16.03). Taxonomic assignments were performed at the phylum, genus, and species levels, and the relative abundance patterns of dominant taxa across these taxonomic ranks are presented in Fig. 5A–C.

At the phylum level (Fig. 5A), a marked increase in the relative abundance of *Pseudomonadota* was observed following the disinfection procedures. In the control group, the proportion of *Pseudomonadota* increased from 44.20% to 72.02%, while in the T2D group it rose from 41.43% to 83.87%. In contrast, *Bacillota* decreased in both groups, declining from 52.26% to 26.90% in the control group and from 56.46% to 15.91% in the T2D group. Reductions were also observed among other low-abundance phyla, including *Spirochaetota* (2.29% to 0.92%; 0.55% to 0.12%), *Actinomycetota* (0.21% to 0.02%; 0.95% to 0.03%), and *Bacteroidota* (0.60% to 0.09%; 0.24% to 0.06%). *Synergistota* and *Fusobacteriota* were no longer detected after treatment, particularly in the T2D.aPDT group, whereas *Mycoplasmatota*, *Patescibacteria*, and *Chloroflexota* remained present at very low relative abundances.

At the genus level (Fig. 5B), pronounced changes in microbial composition were observed after the disinfection procedures. In the control group, the relative abundance of *Paenacidovorax* increased from 4.51% to 32.73%, while *Acinetobacter* increased from 1.02% to 11.25%. In contrast, obligate anaerobic genera such as *Dialister* (24.30% to 6.97%), *Desulfobulbus* (11.29% to 0.03%), and *Anaeroglobus* (7.33% to 0.77%) showed marked reductions. In the T2D group, this pattern was more pronounced, with increases in *Paenacidovorax* (11.82% to 35.53%), *Pseudomonas* (14.11% to 19.03%), and *Stenotrophomonas* (9.49% to 13.38%). Conversely, *Dialister* decreased from 26.33% to 0.19%, *Veillonella* from 12.93% to 1.03%, and *Anaeroglobus* from 2.75% to 0.04%. In addition, genera with higher oxygen tolerance, such as *Ralstonia* (1.05% to 4.48%) and *Variovorax* (1.41% to 3.63%), exhibited increased representation following treatment, particularly in the T2D.aPDT group (Fig. 5B).

At the species level, the dominant species profiles in both groups shifted markedly after the disinfection procedures. In the control group, the relative abundance of *Paenacidovorax caeni* increased from 5.54% to 37.02%, while *Dialister invisus* decreased from 28.65% to 7.88% and *Desulfobulbus oralis* from 13.87% to 0.04%. In the T2D group, *Paenacidovorax caeni* increased from 12.97% to 40.52%, accompanied by increases in *Pseudomonas aeruginosa* (15.40% to 21.25%) and *Stenotrophomonas nitritireducens* (10.42% to 15.26%). In contrast, *Dialister invisus* declined from 25.22% to 0.05%, and *Veillonella parvula* decreased from 10.42% to 0.38% (Fig. 5C).

### Identification of differentially abundant taxa

**Fig. 6:**
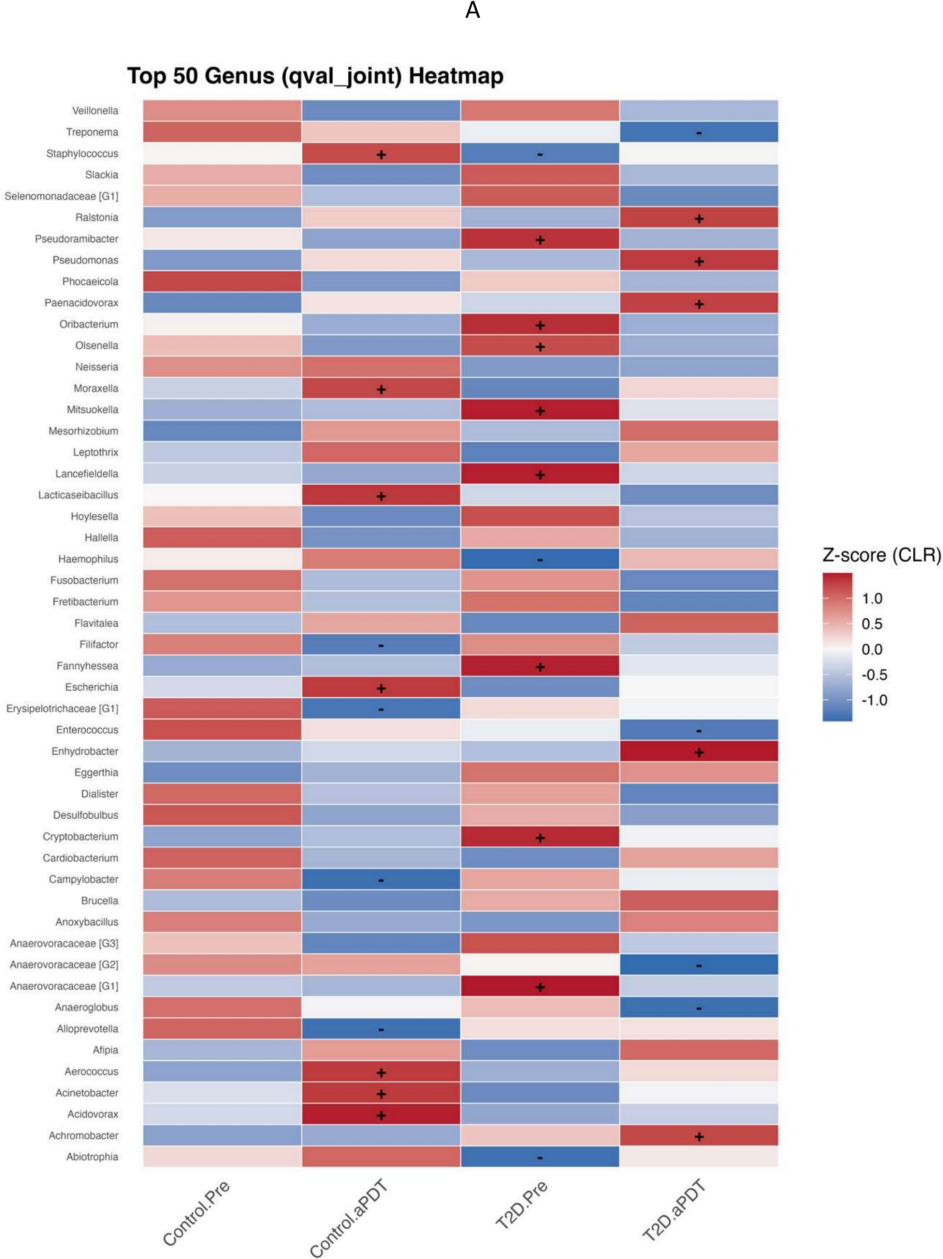

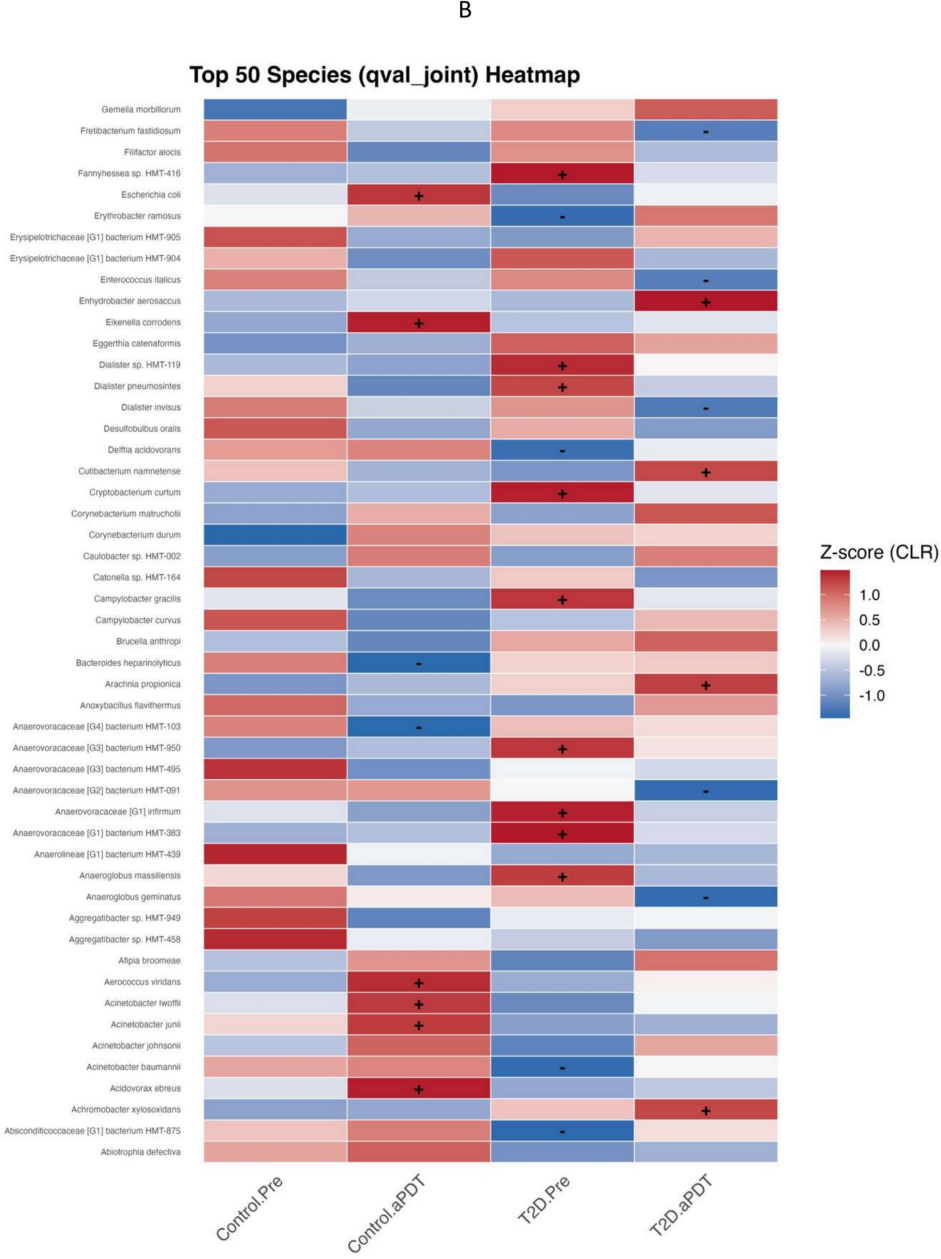
Heatmap of the most abundant bacterial genera (**A**) and species (**B**) showing group-associated differences identified by MaAsLin3 analysis. Color intensity represents centered log-ratio (CLR) Z-scores for each taxon across groups, where red indicates a relative increase and blue indicates a relative decrease in abundance. The symbols “ + ” and “–” denote directionally consistent trends that did not reach statistical significance, corresponding to relative increases or decreases, respectively. Blank cells indicate taxa that were not retained or reported by the MaAsLin3 model for the respective group comparisons

To assess taxonomic differences among samples, a multivariable association analysis was performed using MaAsLin3. This analysis identified genus- and species-level taxa showing group-associated abundance patterns in the root canal microbiota, which are summarized as heatmaps in Fig. 6A and B. Taxa were selected based on the qval_joint criterion, and their CLR-transformed relative abundances are presented as Z-scores across study groups using Control.Pre as the reference group. Positive and negative values indicate relative enrichment or depletion trends, respectively, compared with the reference condition. At the genus level (Fig. 6A), the Control.aPDT group showed positive associations for *Staphylococcus*, *Moraxella*, *Lacticaseibacillus*, *Escherichia*, *Aerococcus*, *Acinetobacter*, and *Acidovorax*, whereas *Filifactor*, *Erysipelotrichaceae [G1]*, *Campylobacter*, and *Alloprevotella* were represented by negative associations. In the T2D.Pre group, *Pseudoramibacter*, *Oribacterium*, *Olsenella*, *Mitsuokella*, *Lancefieldella*, *Fannyhessea*, *Cryptobacterium*, and *Anaerovoracaceae [G1]* showed positive associations. In the T2D.aPDT group, positive associations were observed for *Ralstonia*, *Pseudomonas*, *Paenacidovorax*, *Enhydrobacter*, and *Achromobacter*, while *Treponema*, *Enterococcus*, *Anaerovoracaceae [G2]*, and *Anaeroglobus* exhibited negative associations. At the species level (Fig. 6B), the Control.aPDT group demonstrated positive associations for *Escherichia coli*, *Eikenella corrodens*, *Aerococcus viridans*, *Acinetobacter lwoffii*, *Acinetobacter junii*, *Acidovorax ebreus*, and *Achromobacter xylosoxidans*, whereas *Bacteroides heparinolyticus* and *Anaerovoracaceae [G4] bacterium HMT-103* showed negative associations. In the T2D.Pre group, *Fannyhessea* sp. HMT-416, *Dialister* sp. HMT-119, *Dialister pneumosintes*, *Cryptobacterium curtum*, *Campylobacter gracilis*, *Anaerovoracaceae [G3] bacterium HMT-950*, *Anaerovoracaceae [G1] infirmum*, *Anaerovoracaceae [G1] bacterium HMT-383*, and *Anaeroglobus massiliensis* displayed positive associations. In contrast, *Escherichia coli*, *Delftia acidovorans*, and *Acinetobacter baumannii* were represented by negative associations.

**Fig. 7:**
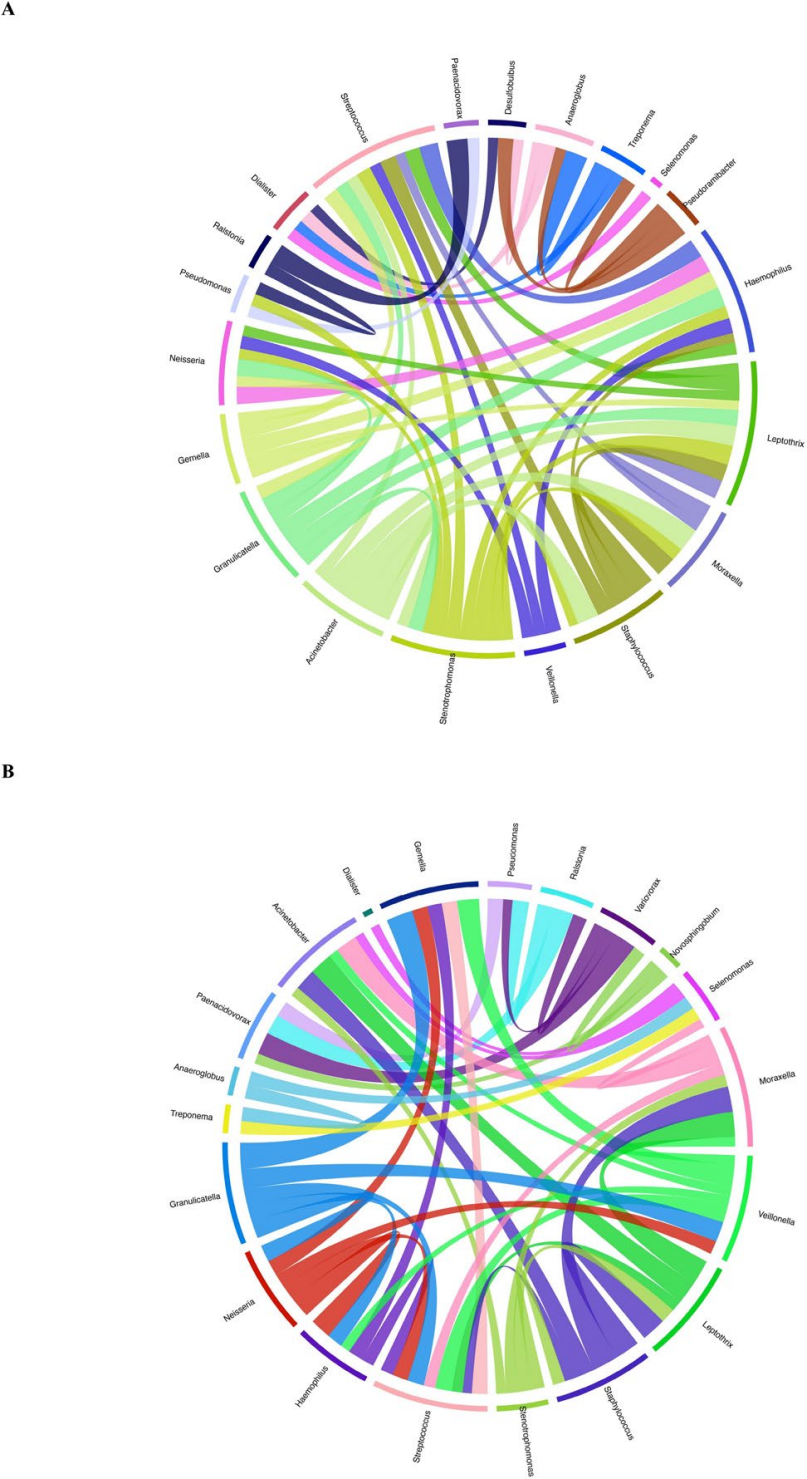

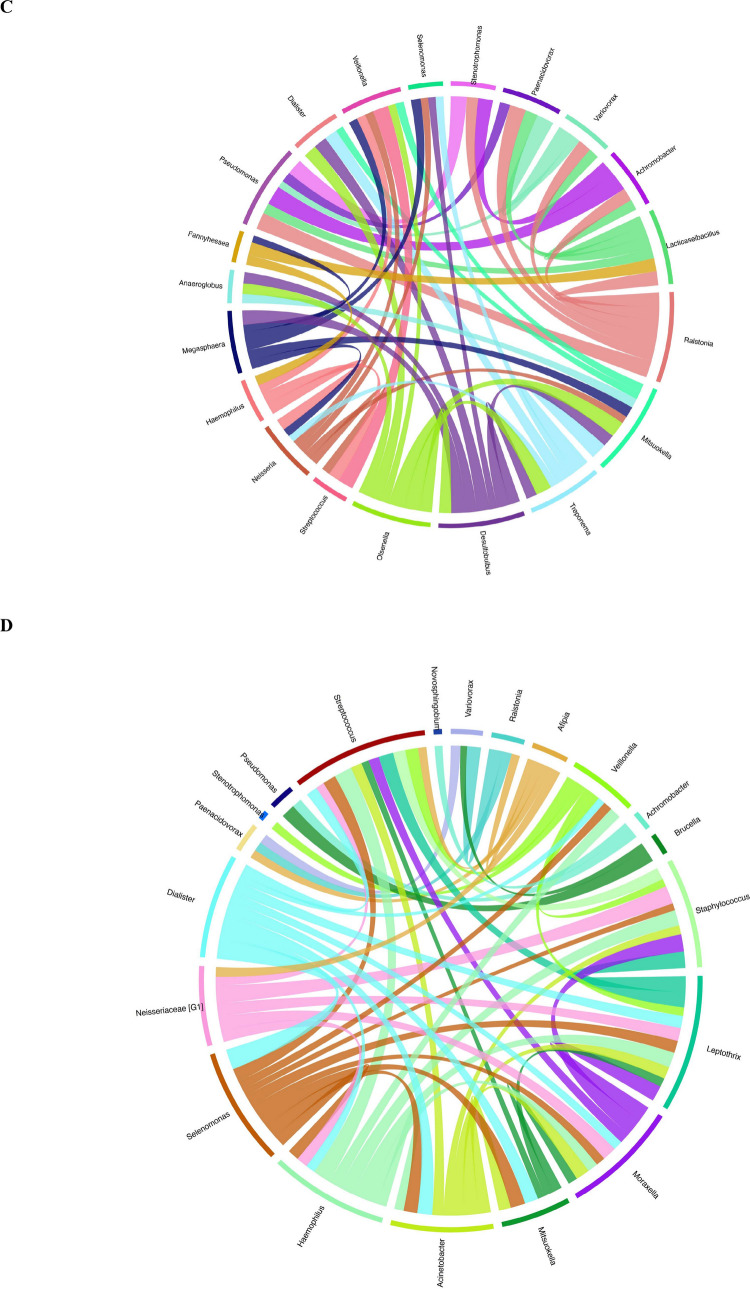
Each Circos plots (**A**–**D**) illustrate the genus-level associations between bacterial taxa and corresponding sample groups. Each band represents a bacterial genus, with the band’s thickness indicating the relative abundance of that genus in the respective group. Panels represent: (**A**) Control.Pre, (**B**) Control.aPDT, (**C**) T2D.Pre, (**D**) T2D.aPDT

In the Circos plots, band thickness reflects the relative dominance of bacterial genera (Fig. 7A–D). In the Control.Pre group (Fig. 7A), the greatest band thicknesses were observed for *Leptothrix* and *Stenotrophomonas*, whereas *Paenacidovorax*, *Desulfobulbus*, *Anaeroglobus*, *Treponema*, *Selenomonas*, and *Pseudoramibacter* exhibited the lowest representation. In the Control.aPDT group (Fig. 7B), dominance was mainly concentrated in *Stenotrophomonas*, *Acinetobacter*, and *Moraxella*. In the same panel, *Paenacidovorax*, *Anaeroglobus*, *Treponema*, *Desulfobulbus*, *Dialister*, and *Selenomonas* were represented by the lowest band thicknesses. In the T2D.Pre group (Fig. 7C), the highest representation was observed for *Moraxella*, *Leptothrix*, and *Veillonella*, whereas *Anaeroglobus*, *Paenacidovorax*, *Desulfobulbus*, *Treponema*, and *Dialister* were present at the lowest levels. In the T2D.aPDT group (Fig. 7D), *Acinetobacter*, *Stenotrophomonas*, *Moraxella*, and *Leptothrix* emerged with the greatest band thicknesses, while *Anaeroglobus*, *Treponema*, *Desulfobulbus*, *Paenacidovorax*, and *Selenomonas* showed the lowest representation.

## Discussion

While the microbial etiology of AP has been partially delineated, the extent to which systemic host conditions modulate the composition of the root canal microbiome and influence the efficacy of endodontic disinfection strategies remains insufficiently defined. This study compared the effects of aPDT combined with chemomechanical preparation on the root canal microbiota in samples obtained from asymptomatic AP cases in healthy individuals and T2D patients Following chemomechanical preparation combined with aPDT, the response of the root canal microbiota to disinfection was evaluated as potentially differing in the presence of diabetes. Considering the globally increasing prevalence of diabetes and its well-documented effects on immune response and oral microbial ecology, these differences in the post-disinfection root canal microbiota should be interpreted within this context.

In this study, the combination of chemomechanical preparation and aPDT resulted in significant structural changes at the β-diversity level in the root canal microbiota of both healthy individuals and patients with T2D. The clear separation between pre- and post-treatment microbial communities suggests that the applied disinfection protocol exerted a selective effect on the root canal ecosystem. Our findings indicate that this effect was primarily associated with a compositional reorganization of the microbial community, characterized by the elimination of low-abundance and niche-associated microorganisms, while the relative abundance patterns of dominant taxa were largely preserved. Previous clinical studies have similarly reported that chemomechanical preparation induces β-diversity-level shifts in the root canal microbiota, reflecting a reorganization of community structure rather than complete microbial eradication, with certain microbial components persisting after treatment [17, 26]. Within this context, the adjunctive use of aPDT may have reinforced the selective pressure imposed by chemomechanical preparation, thereby supporting the observed restructuring of the microbial community [27].

In the present study, 50 ASVs were shared between pre- and post-disinfection samples in healthy individuals, whereas only 21 ASVs persisted across the same time points in patients with T2D.This suggests that the presence of systemic disease may limit the post-treatment ecological stability and taxonomic persistence of the root canal microbiota. Moreover, the substantially lower number of ASVs retained across pre- and post-treatment samples in the T2D group compared with controls highlights a systemic disease-associated reduction in taxonomic persistence following disinfection. Supporting these findings, Sánchez‐Sanhueza et al. [28] reported that individuals classified as ASA II–III exhibited a lower proportion of shared taxa compared to ASA I individuals, with the microbial composition displaying a more individualized pattern. Shook et al. [29] highlighted that hyperglycemia facilitates the development of antibiotic resistance, providing certain microorganisms with a selective advantage in the diabetic environment and enabling them to become more dominant within the microbial community. These associations may reflect contributions from T2D-related systemic factors to reduced microbial ecosystem stability after treatment.

A significant reduction in the richness component of alpha diversity was observed in T2D patients following disinfection procedures, whereas no notable changes in this parameter were detected in healthy individuals. These findings indicate that the microbial response to disinfection procedures varies according to systemic health status. Previous studies [30–32] have reported that hyperglycemia, immune dysregulation, and impaired pulpal microcirculation observed in diabetic individuals contribute to reduced oxygen tension, thereby facilitating the colonization of anaerobic microorganisms. Therefore, the higher baseline abundance of anaerobic bacteria in diabetic individuals may be associated with the more pronounced antimicrobial effect observed following chemomechanical disinfection and adjunctive aPDT; this observation is consistent with the well-documented susceptibility of anaerobic microorganisms to oxidative stress [33, 34]. On the other hand, the preservation of alpha diversity following disinfection in healthy individuals suggests that their oral microbiota may possess greater resilience and ecological stability in response to environmental stressors. This microbial resilience could be associated with a more functionally redundant and competitively balanced community, which resists collapse following external perturbation such as aPDT. Several studies have reported higher levels of salivary bacterial diversity in healthy individuals compared to those with diabetes [32, 35]. It has been reported in the literature that highly diverse microbial communities exhibit functional resistance, which may inhibit the overgrowth of pathogenic species [36]. Within this context, these findings suggest that systemic health status may influence microbial stability.

Following chemomechanical disinfection and aPDT, a similar ecological restructuring was observed in the root canal microbiota of both healthy individuals and patients with T2D. This transformation was characterized, at the phylum level, by the suppression of predominantly anaerobic groups such as *Bacillota*, *Fusobacteriota*, and *Bacteroidota*, accompanied by a relative dominance of the oxygen-tolerant phylum *Pseudomonadota*. The same trend was preserved at the genus and species levels; while obligate anaerobes closely associated with endodontic infections such as *Dialister*, *Veillonella*, and *Desulfobulbus* (*Dialister invisus*, *Veillonella parvula*, *Desulfobulbus oralis*) showed marked reductions, the relative abundance of oxygen-tolerant and metabolically versatile taxa, including *Pseudomonas*, *Stenotrophomonas*, *Acinetobacter*, and *Paenacidovorax*, increased. This consistently observed pattern across taxonomic levels is in agreement with previous reports in the endodontic microbiology literature, which indicate that chemomechanical preparation exerts a fundamental ecological effect by weakening anaerobic dominance within the root canal microbiota [26]. The literature indicates that the microbial shifts observed in the root canal ecosystem following chemomechanical preparation are largely associated with an ecologically driven restructuring resulting from changes in redox conditions. Obligate anaerobic bacteria rely primarily on fermentative metabolism for energy production, rendering them highly dependent on micro-niches characterized by low oxygen partial pressure and reduced redox potential. The combined application of mechanical instrumentation and irrigation disrupts these micro-niches, thereby imposing an environmental pressure that limits the growth and competitive capacity of anaerobic microorganisms. In contrast, the metabolic flexibility of facultative and aerobic bacteria, along with their ability to utilize alternative respiratory pathways, may confer a relative ecological advantage in the post-treatment root canal environment [37, 38]. In this context, the pronounced dominance of the phylum *Pseudomonadota* observed in both groups following aPDT application, together with the concomitant increase in the relative abundance of oxygen tolerant genera and species such as *Paenacidovorax*, *Pseudomonas*, *Stenotrophomonas*, and *Acinetobacter*, particularly *Paenacidovorax caeni*, *Pseudomonas aeruginosa*, and *Stenotrophomonas nitritireducens*, is consistent with an oxygen sensitive compositional shift accompanying the marked reduction in anaerobic populations. It has been previously reported that aPDT, when applied as an adjunct to chemomechanical preparation, may exert a complementary selective pressure contributing to this ecological transition. During aPDT, ROS generated through photosensitizer activation have been shown to exacerbate cellular damage in obligate anaerobic bacteria with limited oxidative defense mechanisms, whereas aerobic and facultative bacteria generally exhibit greater tolerance to oxidative stress In this context, the concomitant reduction in anaerobic populations and the relative enrichment of oxygen-tolerant taxa suggest that the combined application of chemomechanical preparation and aPDT may influence microbial composition not only through direct microbicidal effects but also via a secondary ecological selection mechanism sensitive to oxygen and redox conditions [39, 40].

This study assessed only bacterial communities, while other microbial groups such as viruses, fungi, and archaea were not included. As sampling was performed at a single time point, the temporal dynamics of microbial shifts could not be evaluated. Furthermore, although the duration of disease varied among T2D patients, this parameter could not be incorporated into subgroup analyses, and no data were available regarding host immune responses. In addition, differences in glycemic control, medication use, and oral hygiene status among T2D participants may have influenced the microbial profiles observed, but such data were not available in the current study.

This study demonstrated that the combined application of chemomechanical preparation and aPDT leads to a pronounced structural reorganization of the root canal microbiota in cases of asymptomatic AP. The overall direction of this reorganization was similar in both healthy individuals and patients with T2D. However, in the T2D group, reduced microbial stability was observed in certain metrics related to alpha diversity and taxonomic persistence. These findings suggest that, in the presence of diabetes, the quantitative characteristics of the microbial response following disinfection may differ, while the fundamental ecological effect of the applied disinfection protocol on the root canal microbiota remains unchanged.

## Data Availability

No datasets were generated or analysed during the current study.
